# Personalized biomechanical tongue models based on diffusion-weighted MRI and validated using optical tracking of range of motion

**DOI:** 10.1007/s10237-021-01435-7

**Published:** 2021-03-07

**Authors:** K. D. R. Kappert, L. Voskuilen, L. E. Smeele, A. J. M. Balm, B. Jasperse, A. J. Nederveen, F. van der Heijden

**Affiliations:** 1 Department of Head and Neck Oncology and Surgery, Netherlands Cancer Institute, Antoni Van Leeuwenhoek Hospital, Plesmanlaan 121, 1066 CX Amsterdam, The Netherlands; 2 Department of Robotics and Mechatronics, Faculty of EEMCS, Technical Medical Centre, University of Twente, Enschede, The Netherlands; 3 Department of Radiology and Nuclear Medicine, Amsterdam UMC, University of Amsterdam, Amsterdam, The Netherlands; 4 Department of Oral and Maxillofacial Surgery, Academic Centre for Dentistry Amsterdam and Amsterdam UMC, University of Amsterdam and VU University Amsterdam, Amsterdam, The Netherlands; 5 Department of Oral and Maxillofacial Surgery, Amsterdam UMC, University of Amsterdam, Amsterdam, The Netherlands

**Keywords:** Personalized modeling, Finite element, Tongue, Range of motion, Magnetic resonance imaging, Constrained spherical deconvolution

## Abstract

For advanced tongue cancer, the choice between surgery and organ-sparing treatment is often dependent on the expected loss of tongue functionality after treatment. Biomechanical models might assist in this choice by simulating the post-treatment function loss. However, this function loss varies between patients and should, therefore, be predicted for each patient individually. In the present study, the goal was to better predict the postoperative range of motion (ROM) of the tongue by personalizing biomechanical models using diffusion-weighted MRI and constrained spherical deconvolution reconstructions of tongue muscle architecture. Diffusion-weighted MRI scans of ten healthy volunteers were obtained to reconstruct their tongue musculature, which were subsequently registered to a previously described population average or atlas. Using the displacement fields obtained from the registration, the segmented muscle fiber tracks from the atlas were morphed back to create personalized muscle fiber tracks. Finite element models were created from the fiber tracks of the atlas and those of the individual tongues. Via inverse simulation of a protruding, downward, left and right movement, the ROM of the tongue was predicted. This prediction was compared to the ROM measured with a 3D camera. It was demonstrated that biomechanical models with personalized muscles bundles are better in approaching the measured ROM than a generic model. However, to achieve this result a correction factor was needed to compensate for the small magnitude of motion of the model. Future versions of these models may have the potential to improve the estimation of function loss after treatment for advanced tongue cancer.

## Introduction

The incidence of tongue cancer is rising worldwide, accounting for almost 20% of all head and neck cancers (Tota et al. 2017; UK Cancer Research 2019). Locally advanced tongue cancer is usually treated by surgery and/or chemoradiation, which may have a serious impact on the mobility of the tongue due to surgical defects and/or radiation-induced fibrosis. This often leads to difficulties with speech, mastication, and swallowing (Konstantinović and Dimić 1998; Kreeft et al. 2009b). The choice between surgical and organ-sparing treatment is dependent on expected function loss after treatment, which is difficult to predict (Kreeft et al. 2009a). The prediction of the expected function loss would be of great benefit for the decision-making process shared between physician and patient. Biomechanical modeling of the tongue would be a logical next step in the process of the prediction of functional loss.

The biomechanics of the tongue, however, are complex (Bressmann et al. 2004; Matsui et al. 2007; Kreeft et al. 2009a). The tongue consists of four extrinsic and four intrinsic muscles, which interdigitate and seem to follow a strict pattern (Sanders and Mu 2013). Although we know that all muscles, except for the palatoglossus muscle, are innervated by the hypoglossal nerve (Takemoto 2001; Mu and Sanders 2010), the complex neural strategies that are required for shaping the tongue during speech and mastication are currently unknown (Slaughter et al. 2005; Van Alphen et al. 2017). Moreover, the tongue shape varies between individuals, and knowledge about anatomical variations in muscle structure does not yet exist (Stone et al. 2018). Because of the difficulties in obtaining the right mechanical properties and simulating the viscoelastic nature of the tongue, mechanical properties are often approached by a hyperplastic model using ex vivo data (Buchaillard et al. 2007; Hermant et al. 2017; Kappert et al. 2019b, 2021).

Despite the challenges, biomechanical finite-element (FE) models have shown to be a promising method to predict functional loss after treatment in their current form (Buchaillard et al. 2007; Hermant et al. 2017; Kappert et al. 2019b). However, these FE models are generally generic and are therefore unable to predict functional loss on an individual level and should be personalized.

One way of creating personalized FE models is by morphing of a generic FE model to a subject-specific situation (Couteau et al. 2000; Sigal et al. 2008). Previous work has shown that this morphing can be driven by imaging data such as anatomical slices (Fernandez et al. 2004), computed tomography (Bucki et al. 2010; Grassi et al. 2011), and MRI (Barber et al. 2007; Bijar et al. 2016). Alternatively, personalized models can also be constructed by embedding mesh and muscle structures in a FE model that is generated according to the shape of the mesh. (Nesme et al. 2009; Sánchez et al. 2017). If muscles are, however, included in personalized models, the morphing should not only be driven by the outline of anatomical structures or meshes, but also the internal structure of the muscle, such as the muscle fiber directionality.

This muscle fiber directionality can be measured by exploiting the possibilities of diffusion-weighted MRI (Van Donkelaar et al. 1999). Using diffusion-sensitizing gradients, it is possible to encode MR images with diffusion information along a certain direction. As the diffusivity of water is higher along muscle fibers than perpendicular to them, it is possible to reconstruct the fiber orientation using the diffusion tensor. In fiber tracking or tractography, fiber tracks are computed from these fiber orientations, (Basser et al. 2000), easing the visualization of the tongue musculature (Napadow et al. 2001; Shinagawa et al. 2006; Heemskerk et al. 2010; Ye et al. 2015). These tracks have even been used as an input for biomechanical models of the tongue (Mijailovich et al. 2010). Despite this potential of DTI, it is unable to resolve crossing or merging muscle fibers of the tongue. Recently, a diffusion-weighted MRI technique called constrained spherical deconvolution (CSD), which can resolve the interdigitating muscle fibers of the tongue in vivo, was applied to the tongue (Voskuilen et al. 2019). This technique enables us to reconstruct the tongue muscle architecture of the individual more accurately.

The goal of the current work was to create personalized biomechanical models of the tongue by using CSD MRI. As manual embedding of all the fibers of this muscle architecture in the FE model would be very laborious, it is hardly feasible. This motivated us to use automated methods to embed these fibers. While CSD MRI is superior in resolving crossing and merging fibers, the high noise level resulted in relatively low-quality reconstructions of the tongue musculature of a single subject. We, therefore, proposed to use a population average or atlas of the tongue muscle architecture (Voskuilen et al. 2018), which is more resistant to noise and artifacts. By mapping the tongue of an individual to the atlas tongue, we hypothesized that the segmented fiber tracks of the atlas could be morphed back to an individual’s space and that, subsequently, from these segmented fiber tracks a personalized biomechanical model could be created. The effect of this personalization step was evaluated by comparing models with both personalized and generic muscles bundles to the predicted range of motion (ROM) of the tongue measured in vivo using 3D optical tracking (Kappert et al. 2019a).

## Methods

**Fig. 1:**
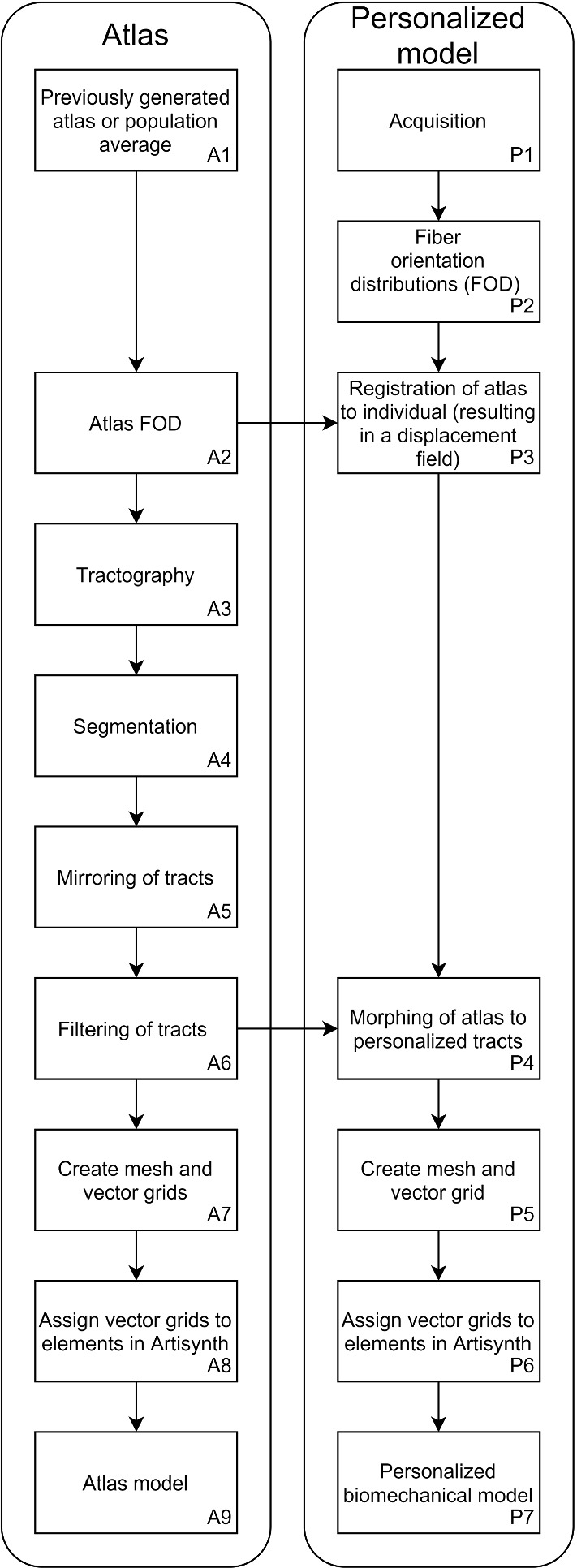
A flow chart of the steps required to create an atlas-based (*A1–9*) and a personalized model (*P1–7*)

The following section covers the characteristics of volunteers and the measurement of their ROM. Hereafter, the creation of the personalized biomechanical models is described, which is summarized in Fig. 1. This figure helps the reader to follow the steps that are needed for creating the atlas model (Fig. 1A1–9) and the personalized model (Fig. 1P1–7) as the method section covers both models at the same time. Finally, the ROM predicted by these biomechanical models and the atlas were compared to the measured ROM.

### Volunteers & ROM measurement

A total of ten healthy volunteers were included with a mean age of 61 years (range: 56–71; seven men) to match the same age group of most tongue cancer patients. Volunteers with steel braces or any other contra-indication to an MRI scan were excluded.

The ROM of the tongue was obtained by optical tracking of a marker on the tip of the tongue using a 3D camera. The volunteers were asked to perform four different tongue movements: left, right, down, and protrusion as described in the paper by Kappert et al. (2019a). The up-movement was left out since it was proven to be unreliable. Written informed consent was obtained from all volunteers before inclusion. This study was approved by the medical ethical committee of the Netherlands Cancer Institute (ref: N17BTM).

### CSD MRI acquisition and processing

The volunteers were scanned in a 3 T Philips MRI scanner (Philips Healthcare, Best, The Netherlands) using a neurovascular coil according to the CSD scan protocol by Voskuilen et al. (2019) (Fig. 1P1). The raw diffusion-weighted images were acquired using the following parameters: single-shot spin-echo echo-planar imaging; echo-train length 25; repetition time: 3.4 s; echo time: 60 ms; two repetitions with opposing phase-encoding directions; number of signal averages: 1; fat suppression: spectral presaturation with inversion recovery and slice-selection gradient reversal; field-of-view: 192 by 156 by 84 mm; voxel size: 3 mm isotropic; b-value: 700 s/mm^2^ along 64 directions evenly spaced over a hemisphere and optimized for gradient load; total scan time: 10 min.

Subsequently, the noise of the diffusion-weighted images was reduced using the method of Veraart et al. (2016). Using FSL, a software library for diffusion MRI (Smith et al. 2004), the diffusion-weighted images were corrected for distortions caused by B_0_-inhomogeneity, eddy currents from the diffusion-encoding gradients, and rigid motion (Andersson and Sotiropoulos 2016). For all subjects, masks of the tongue were created by manual delineation in ITK-Snap (Yushkevich et al. 2006). In MRtrix3 (Tournier et al. 2012), the corrected diffusion-weighted images were upsampled to a resolution of 1.5 mm isotropic using a b-spline interpolation. For each volunteer, we estimated a CSD response function, which corresponds to the diffusion signal of a single fiber population (Tournier et al. 2013). By deconvolving, using CSD, the corrected diffusion-weighted images with this response function (Tournier et al. 2007), fiber-orientation distribution (FOD) maps were calculated up to a maximum spherical harmonic degree of 8 (Figs. 1P2, 2).

**Fig. 2:**
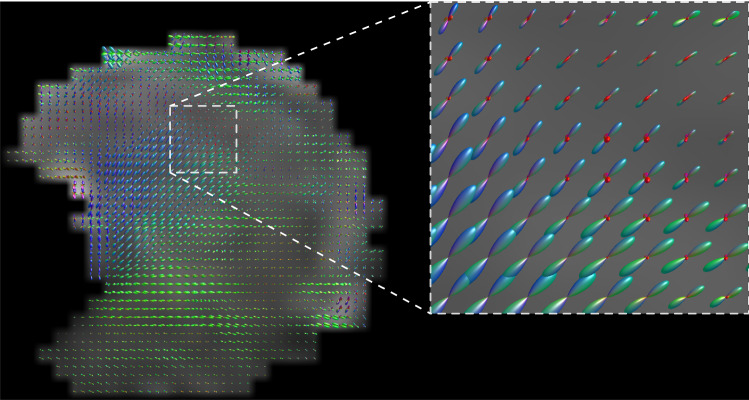
Midsagittal view of an fiber-orientation distribution (FOD) map of volunteer 2. In the inlay, the map is enlarged so the individual FODs can better be appreciated. The FODs are colored according to their direction: red for right–left; green for anterior–posterior; and blue for feet–head

Finally, using symmetric diffeomorphic registration based on the FOD maps (Raffelt et al. 2011), displacement fields were calculated from each volunteer to the tongue muscle atlas described in Voskuilen et al. (2018) (Fig. 1P3). This atlas is a population average of ten volunteers different from those included in this study (mean age of 25.5 years; four female). The atlas has a voxel size of 1.5 mm isotropic after up sampling. The registration error between the FOD maps of the individuals and that of the atlas were quantified by the L_2_-norm and the angular correlation coefficient (Raffelt et al. 2012a). The calculated displacement fields were used later in this work to morph the generical biomechanical model of the atlas.

### Fiber tracking and filtering

Although it would be possible to compute a 3D vector field (required to build a biomechanical model) from the FOD maps directly, CSD-based fiber tracking was first performed on the atlas (Tournier et al. 2010) (Fig. 1A3). Fiber tracking ignored many spurious vectors, and the segmentation of streamlines was less time-consuming than segmentation of vectors. For this global fiber tracking, the following parameters were used: step size: 1.5 mm; angular threshold: 15°; FOD peak threshold: 10% of the largest peak; maximal length: 100 mm; minimal length: 10 mm; number of seed points: 10,000 randomly placed within the mask. In TrackVis (Wang and Benner 2007), the fiber tracking was manually segmented into the following muscle tracts: genioglossus, geniohyoid, hyoglossus, inferior longitudinal, superior longitudinal, transverse, and vertical muscles (Figs. 1A4, 3). These segmentations were subsequently checked by a head-and-neck surgeon. The styloglossus muscle could not be distinguished from the inferior longitudinal and was therefore not included (Voskuilen et al. 2019).

**Fig. 3:**
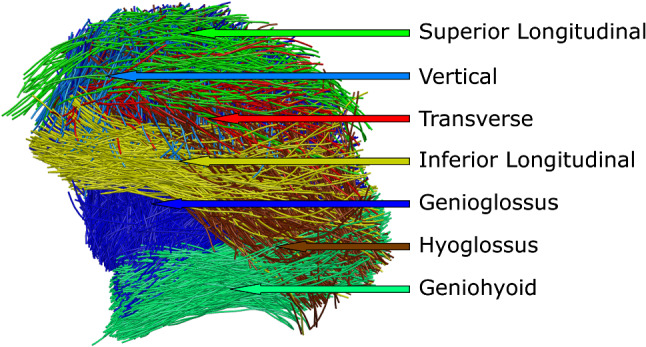
Side view of the segmented fiber tracts of the atlas

In MATLAB R2019a (Mathworks, Natick, MA), these atlas tracks were mirrored in the midsagittal plane to ensure the symmetry of the atlas (Fig. 1A5). To remove faulty tracks, while preserving the muscle shape, the muscle tracts were filtered using the criteria shown in Appendix I (Fig. 1A6). These criteria were chosen empirically based on the reduction in outliers and the expected curvature obtained from earlier anatomical research (Takemoto 2001).

For each volunteer of the study group, the displacement fields obtained from the registration earlier were used to morph the filtered tracts from the atlas into personalized tracts (Fig. 1P4). The personalized tracts may have been rotated during morphing and were therefore reoriented, based on the orientation of the muscles before morphing.

### FE model construction

**Fig. 4:**
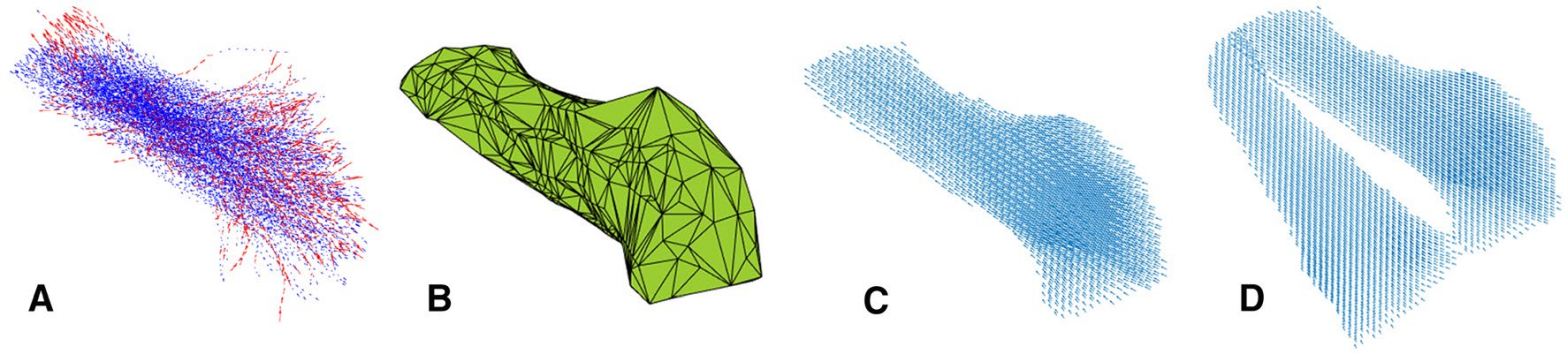
Tracks (blue) and filtered tracks (red) from the inferior longitudinal muscle (**a**); the convex hull enclosing these tracks (**b**); a uniformly distributed vector field based on the direction of the tracks within the convex hull (**c**); and vector fields of both left and right muscles (**d**)

**Fig. 5:**
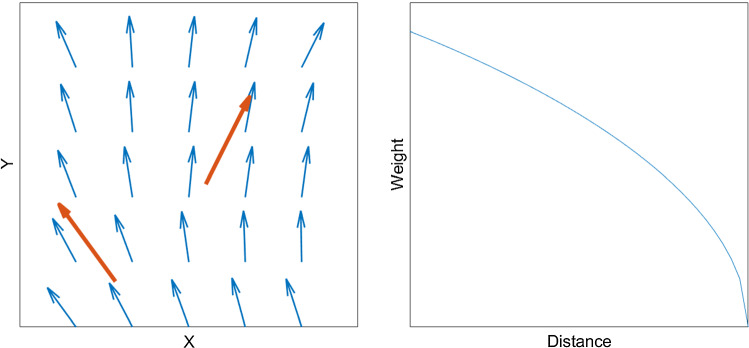
Direction of ${\mathbf{n}}_{{{\text{grid}}}} \left( {\mathbf{x}} \right)$ (small blue arrows) is determined by nearby ${\mathbf{n}}_{{{\text{track}}_{{\text{i}}} }}$ (large red arrows) by means of inverse distance interpolation [Eq. (1)] visualized in the right figure

The tracks of both the atlas model and personalized models were converted into vector fields of muscle fiber direction (Fig. 1P5, A7), using the following steps. For each muscle, a convex hull was calculated that enclosed the filtered tract (Fig. 4a, b). These convex hulls were filled with a uniformly distributed grid of vectors, where the direction of these vectors was determined by an inverse distance interpolation of nearby tracks [Figs. 4c, 5; Eq. (1)]. This was done for both left and right muscles independently if applicable (Fig. 4d).with

$${\varvec{n}}_{{{\text{grid}}}} \left( {\varvec{x}} \right) = \mathop \sum \limits_{i} \left( {1 - \sqrt {\frac{{d\left( {{\varvec{x}},{\text{track}}_{i} } \right)}}{{\max_{{\varvec{x}}} (d\left( {{\varvec{x}},{\text{track}}_{i} } \right))}}} } \right){\varvec{n}}_{{track_{i} }}$$

1 $${\varvec{n}}_{{{\text{grid}}}} \left( {\varvec{x}} \right) = \frac{{{\varvec{n}}_{{{\text{grid}}}} \left( {\varvec{x}} \right)}}{{{\varvec{n}}_{{{\text{grid}}}} \left( {\varvec{x}} \right)}}$$

Equation (1) ${\varvec{n}}_{{{\text{grid}}}} \left( {\varvec{x}} \right)$ the uniformly distributed grid of vectors within the convex hull, ∑_*i*_ the sum over all vectors and ${\varvec{n}}_{{{\text{track}}_{i} }}$, the original tracks. $d\left( {{\varvec{x}},{\text{track}}_{i} } \right)$ is the distance of vector ${\varvec{x}}$ to ${\text{track}}_{i}$.

The genioglossus muscle was divided into an oblique and horizontal part based on the estimated position of the short tendon (Sanders and Mu 2013). With all tracks combined, a convex hull was generated to create a mesh of the tongue. In Meshlab (Cignoni et al. 2008), the HC Laplacian filter (Vollmer et al. 1999) was used to smoothen the mesh. Attachment points for the mandible and hyoid bones were determined based on the endpoints of the extrinsic muscle tracts from the atlas model.

Using ArtiSynth (Lloyd et al. 2012)—a platform for combined multibody and FE modeling—the muscle vector fields and tongue meshes of the atlas and the ten volunteers were integrated into a biomechanical model (Figs. 1P6, A8, 6) using the following steps, which are similar to those described by Kappert et al. 2019b. The surface mesh of the tongue, obtained from the previous step, is embedded into a FE model consisting of 16 mm^3^ cubic hexahedral elements, generated to match the shape of the surface mesh. The benefit of only using cubic elements is that they can easily be removed or added, which is an essential feature to simulate surgical resections on personalized biomechanical models in the future (Kappert et al. 2019b). The attachment points to the hyoid and mandible were simulated by making FE nodes non-dynamic.

**Fig. 6:**
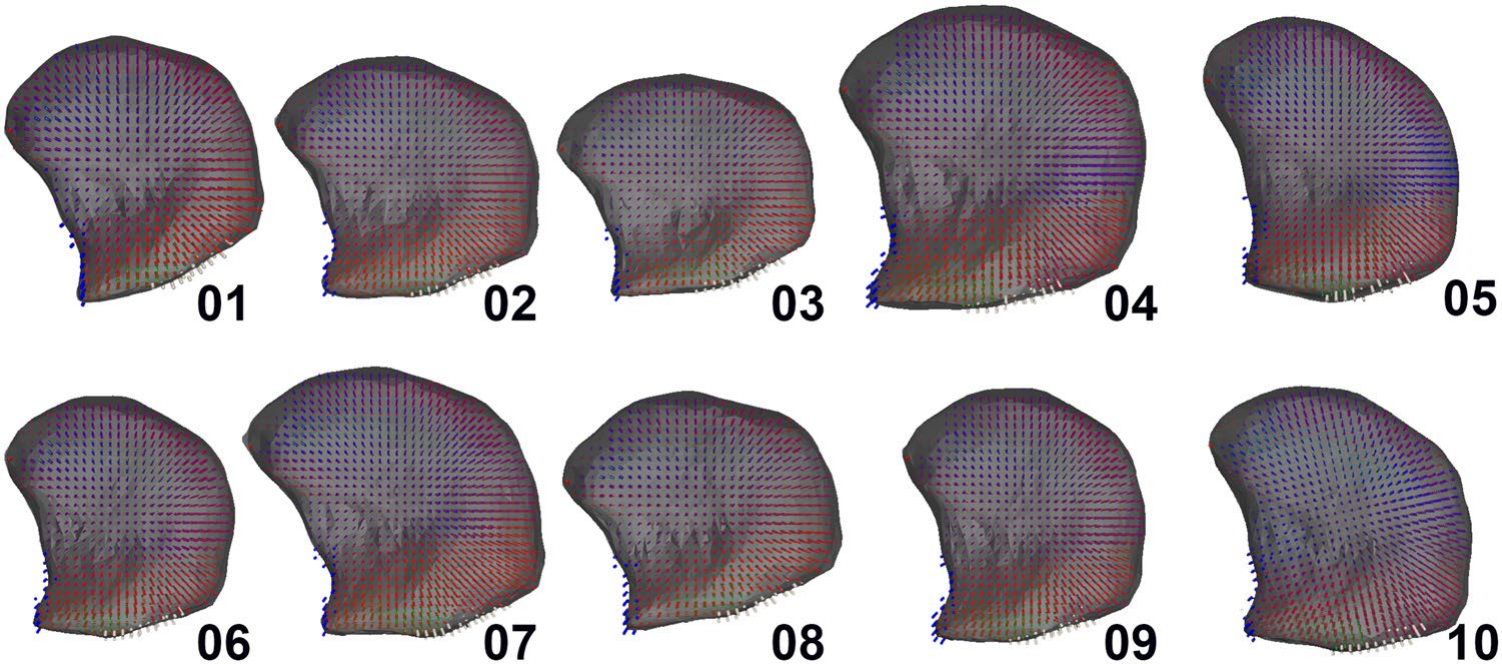
Sagittal section view of personalized FE tongue models of the ten healthy volunteers. The direction of force of the muscle elements has been color-coded: anterior–posterior in red; right–left in green; and feet–head in blue. Bone attachment points are visualized as floating point outside the mesh. The mandible attachment points are visualized in blue and those of the hyoid bone in white

Based on previous work (Buchaillard et al. 2009), an incompressible Moony–Rivlin material was chosen for mechanical properties of the tissue:

$$W = C_{10} (I_{1} - 3) + C_{20} (I_{1} - 3)^{2} + {\upkappa }\left( {\ln J} \right)^{2}$$

where $I_{1}$ is the first invariant of the left Cauchy–Green deformation tensor, $C_{10}$ and $C_{20}$ stiffness parameters equal to 1037 and 486 Pa, ${\upkappa }$ = 100 × C_10_ the bulk modulus to obtain a Poisson’s ratio close to 0.5, and *J* is the determinant of the deformation gradient. The stiffness parameters are obtained from a fresh cadaver in (Gerard et al. 2005) and later scaled by a factor of 5.4 in (Buchaillard et al. 2009) to match in vivo measurements. C_10_ is equal to 1037 Pa, C_20_ equal is to 486 Pa, and the other parameters are all zero. Rayleigh damping coefficients of *α* = 40 s^−1^ and *β* = 0.03, and a density of 1040 kg/m^3^ were used, comparable to those used by Buchaillard et al. (2009), Stavness et al. (2012) and Kappert et al. (2019b).

Muscle contraction was simulated using “muscle material” in ArtiSynth. With this technique, when a muscle bundle is activated, it applies external stresses on the elements associated with the muscle bundles, in addition to the regular tissue material (Lloyd et al. 2012). The transversely isotropic properties of the muscles were included using ArtiSynth’s “Muscle Material” interpretation of the method by Blemker et al. (2005):

$$\sigma \left( \lambda \right) = \sigma_{\max } \left( {\alpha f_{{{\text{act}}}} \left( \lambda \right) + f_{{{\text{pass}}}} \left( \lambda \right)} \right)\left( {\frac{\lambda }{{\lambda_{{{\text{opt}}}} }}} \right)$$

with $\sigma_{\max }$ the max isometric stress in the muscle, $\alpha$ the normalized activation level, $\lambda$ the stretch along fiber, $\lambda_{{{\text{opt}}}}$ the optimal fiber stretch, $f_{{{\text{act}}}}$ the active force–length relationship, and $f_{{{\text{pass}}}}$ the passive.

### Simulation and analysis

Inverse simulation, provided by ArtiSynth (Lloyd et al. 2012; Stavness et al. 2012), was used to instruct the tongue tip of the personalized FE models to consecutively move to a point anterior, inferior, left, and right of its initial location. The predicted ROM was defined as the distance from the initial location to maximal deflection in one of the instructed directions.

The tongue can reach strain values of 200% for elongation and 160% for contraction (Napadow et al. 1999). Using the current biomechanical model, it is not possible to simulate these magnitudes of deformation. As all model simulations use the same constitutive models, tissue properties, and FE generation technique, the effect thereof is considered to be constant for all models. To evaluate the differences between the FE models with personalized muscle bundles and those using generic muscle bundles (the atlas model), we will therefore focus on the relative differences between individuals and not on the absolute values. In order to compare the simulations to the measured ROM, a scaling factor is needed to compensate for the reduced magnitude of motion of the model. The reduced magnitude of motion will be different depending on the movement direction, and therefore, four scaling factors were calculated and applied to all simulations equally. To make sure outliers would not affect the scaling factors, these factors were determined by an iterative process to achieve the maximum number of predicted ROMs (${\text{ROM}}_{{{\text{pred}}}} \left( {i,j} \right)$) that were within the CI (${\text{ROM}}_{2\sigma }$) of the measured ROM (${\text{ROM}}_{{{\text{meas}}}} \left( {i,j} \right)$) [Eq. (2)].

2 $$S_{i} = \mathop {\text{arg max}}\limits_{{S_{i} }} \mathop \sum \limits_{j = 1}^{10} \left[ {\left| {S_{i} \cdot {\text{ROM}}_{{{\text{pred}}}} \left( {i,j} \right) - {\text{ROM}}_{{{\text{meas}}}} \left( {i,j} \right)} \right| < {\text{ROM}}_{{2{\upsigma }}} } \right]{\text{, where [}}x] = \left\{ {\begin{array}{*{20}c} {0{\text{ if }}x = {\text{false}}} \\ {1{\text{ if }}x = {\text{true}}} \\ \end{array} } \right.$$

in which

$i = 1, \ldots ,4$ Index for the 4 different movements.

$j = 1, \ldots ,10$ Index for the 10 volunteers from the study group.

$${\text{ROM}}_{{{\text{pred}}}} \left( {i,j} \right)$$ Predicted ROM.

$${\text{ROM}}_{{{\text{meas}}}} \left( {i,j} \right)$$ Measured ROM.

$S_{i}$ The scaling factor applied to predicted ${\text{ROM}}_{{{\text{pred}}}} \left( {i,j} \right)$.

${\text{ROM}}_{2\sigma }$ Twice the standard deviation of the measured ROM (i.e., 6 mm).

Equation (2) Optimization of the scaling factor such that the number of predictions ${\text{ROM}}_{{{\text{pred}}}} \left( {i,j} \right)$ within the bounds ${\text{ROM}}_{{2{\upsigma }}}$ of the measured ROM (${\text{ROM}}_{{{\text{meas}}}} \left( {i,j} \right)$) is maximized.

The predicted ROM was compared to the in vivo measured ROM of the individual on which the personalized model was based. To show the benefit of personalization, also the Atlas model (essentially a generic model) will be compared with the measured ROM. Only when the personalized models perform better than the atlas, we can conclude that personalization improves the ROM prediction.

Previously, the precision of the ROM measurements, quantified by the standard deviation, was determined to a range from 2.3 to 3.2 mm (Kappert et al. 2019a). We, therefore, assumed a precision of 3 mm (3.2 mm rounded off) for all ROM measurements. If a predicted ROM fell within the 95% confidence interval (CI), i.e., within two times the standard deviation, we judged the measurement to be correct.

## Results

Visually, the FOD maps were well aligned to the atlas. The error in alignment or registration error was quantified by the L2-norm and the angular cross-correlation. The mean L_2_-norm between the FOD maps and the atlas was 0.302 (SD 0.030). The mean angular correlation coefficient was 0.634 (SD 0.057).

**Fig. 7:**
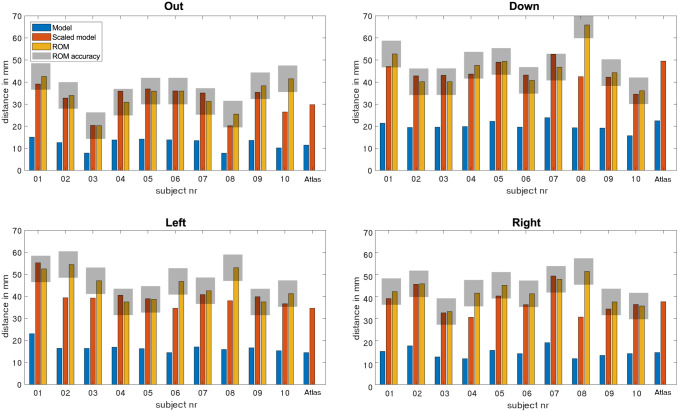
Range of motion (ROM) in mm for the ten healthy volunteers (01–10) and the Atlas (Generic model), for protrusion, and the down, left, and right movements. The predicted ROM of the personalized and atlas (generic model) is given in blue, the scaled predicted ROM in orange, and the measured ROM in yellow. The grey box depicts the interval of two times the standard deviation of the measured ROM within which the predicted ROM values of both atlas and personalized models are assumed to be accurate

In Fig. 7, the distances for specific tongue movements of both the measured ROM and predicted ROM are shown for all ten subjects. For the predicted ROM, both the scaled and non-scaled movements are shown. The scaling factors are 2.6 for protrusion, 2.2 for down, 2.4 for left, and 2.6 for right. Protrusion and down movements show the best agreement between predictions and measurements, as nine out of ten (90%) predicted ROM’s are within the CI. Eight movements (80%) to the right were predicted to be within the CI, but for the movement to the left, only six (60%) were predicted to be within the interval. In total, 32 out of the 40 predictions (4 movements, 10 volunteers) from the personalized models were within the CI. The largest disagreements between the prediction and in vivo measurement were found in subject 08. The atlas model is the same for every subject which is depicted more clearly in the following table.

In Table 1, the percentual differences of the models with the measured ROM are shown for every subject. The model that approaches the measured ROM better differs between subject and movement, but a majority of the measurements are approached better using the personalized model. The mean percentual difference per movement shows that for all movements the difference with the measured ROM is lower for the personalized models.

**Table 1:** Percentual difference between the personalized model or Atlas (generic model) and the measured ROM per subject

Movement	Model	01 (%)	02 (%)	03 (%)	04 (%)	05 (%)	06 (%)	07 (%)	08 (%)	09 (%)	10 (%)	Mean (%)
Out	Personalized	8	4	1	16	3	0	12	21	8	36	11
	Atlas	30	12	47	4	17	17	5	17	22	28	20
Down	Personalized	11	7	7	8	1	6	12	35	5	4	10
	Atlas	6	23	23	4	0	21	6	25	12	37	16
Left	Personalized	5	28	17	8	1	26	4	28	6	11	13
	Atlas	34	36	27	8	10	26	19	35	8	16	22
Right	Personalized	8	0	2	27	11	12	3	40	9	2	11
	Atlas	11	18	14	9	17	9	21	27	0	6	13

The last column shows the mean percentual difference

**Fig. 8:**
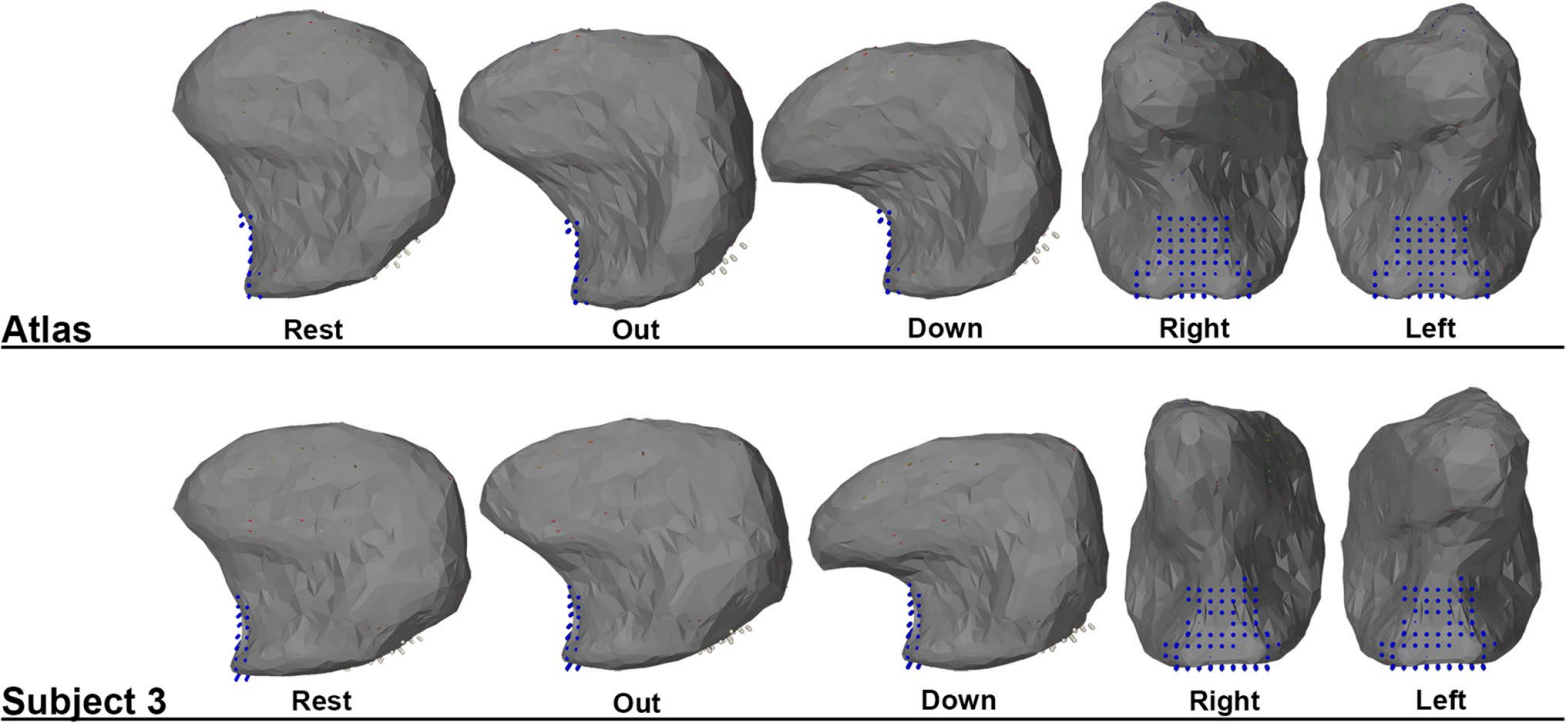
An example of the maximum range in the ROM prediction for protrusion, down, left, and right using the personalized model of subject 1, 10 and the atlas

In Fig. 8, the atlas model and the personalized model of subject 3 are shown within the ArtiSynth environment. For the four simulated movements, the maximal extension is shown. Subject 3 demonstrated a ROM that in 3 out of 4 movements could not be predicted using the atlas, but could be using the personalized model. The movement of the atlas, relative to its rest state, looks larger in most directions than the personalized model as confirmed by the bar charts in Fig. 7. Also, the tongue moves more upwards during the right movement. The magnitude of the movements of both models is smaller than what would be expected from a real tongue.

## Discussion

This study was a first approach to combine CSD MRI and FE modeling to create personalized biomechanical models of ten healthy volunteers. The results show that, after applying a correction factor to the simulations, the personalized models were comparable to the measured ROM in 80% of the cases, whereas the atlas model was only comparable in 50% of the cases. For every individual movement, the personalized models also performed better than the atlas model predicting up to 90% of the down movements correctly. This confirms that using muscle bundles based on CSD in an FE model of the tongue contributes significantly to the personalization of a biomechanical tongue model.

Although the downward movement of subject 8 with a relatively small tongue was exceptionally high, this measurement was confirmed to be correct upon reviewing the images from the 3D camera. The model was not able to reproduce this large ROM, which may indicate that a large ROM is not only a result of differences in tongue muscle morphology. The ROM may also have been affected by other quantities that were not accounted for, such as the number of motor units or the stiffness, anisotropy, and density of the tissue.

In the posterior part or base of the tongue, breathing motion impaired the tracking of the superior longitudinal and transverse muscle (Voskuilen et al. 2019). Although these muscle tracts were filtered less vigorously, this could not resolve the absence of muscle tracts. Fortunately, the effect of the absence of these tracks is expected to be minimal, as the simulation of the ROM is less dependent on the musculature of the posterior tongue. Other artifacts, such as those caused by ferromagnetic crowns, resulted in signal voids in the diffusion-weighted images and therefore gaps in the tractography of the tongue. As the biomechanical models were based on the atlas, where such gaps are not present, we assume these artifacts that occurred in individual data sets would cause minimal errors in the personalized models.

The genioglossus and geniohyoid muscles form one large continuous fan of tracks. As described in the literature, some FE models divide the genioglossus into an anterior, middle, and posterior part (Harandi et al. 2014; Wu et al. 2014; Dabbaghchian et al. 2016; Hermant et al. 2017), while others separate the genioglossus into a horizontal and an oblique subdivision (Mu and Sanders 2010; Honda et al. 2013; Sanders and Mu 2013). We chose the latter because the location of the short tendon could be inferred from our atlas and could, therefore, be used as an anatomical marker to split the genioglossus into two. As the styloglossus could not be distinguished from the inferior longitudinal muscle in the fiber tracking, the styloglossus was omitted from the model. The effect on our simulations was expected to be limited since the styloglossus is mainly involved in retracting the tongue and swallowing.

Similar to CSD in the brain, the apparent fiber density could be derived from CSD in muscles, which should in principle relate to muscle strength (Miller et al. 2002; Raffelt et al. 2012b). Therefore, incorporating this apparent fiber density into our biomechanical models might improve the ROM predictions. However, since CSD MRI in the tongue is subject to higher noise levels and more motion artifacts than for example in the brain, in our opinion, the apparent fiber density can currently not be quantified accurately. We, therefore, assumed that the vectors describing the muscle direction were equally distributed within the area of specific muscles.

While large parts of the methods were automated, some key elements were still done manually. For the atlas, the segmentation of the fiber tracts and the subsequent filtering were done manually. Techniques to automate these segmentation steps are not matured yet, and therefore, manual input is still needed. For the personalized models, only the initial masks were manually delineated. After this step, the models could be processed without manual interference.

In this study, we mirrored the atlas to make it symmetric. However, by applying the displacement fields to create personalized biomechanical models, asymmetry was reintroduced. In this study, the orientation of the personalized biomechanical models was based on the former position of the tracks within the atlas. This leads to small lateral asymmetry in the distribution of muscles. An alternative method would be to label based on its new midline. However, determining the exact midline remains challenging, and without a gold standard, there is no way to determine which method is best.

Similar to previous work (Kappert et al. 2019b), we used hexahedral cubic elements with embedded muscles and mesh for the FE model, which do not optimally represent the shape of a surface. As stated in the previous work, the effect of this method on the mobility of the model is minimal. The choice for this embedded design was made so that in the future the virtual surgery method introduced in the aforementioned study can be used in combination with the personalization proposed in the current study.

In this study, optical tracking of the tongue tip was used to determine the ROM. Not only the tongue but also the mandible and hyoid bone assist the tongue tip in reaching the desired position. How much influence these structures have on the tongue ROM depends on the anatomy, innervation, and brain-muscle control. This influence had not been measured and, instead, a marker on the mandible was used to compensate for the movement of the mandible (Kappert et al. 2019a). This marker may, however, not always reliably compensate for all complex movements, and an error should be expected in the measured ROM. Predicted ROMs were therefore judged on whether they fell within the CI of this error. This CI was relatively wide and might, therefore, have hampered the correct judgment of small variations between the predicted ROM and measured ROM.

In the biomechanical models, the magnitude of the predicted ROM was much smaller than that of the measured ROM which, therefore, had to be scaled in order to be compared with the measured ROM. Incorporating movement of adjacent connected structured such as the hyoid bone might improve the range. Also, the mechanical properties were based on the model of Buchaillard et al. (2009), which uses stiffer material properties than those originally measured in a cadaver study, to simulate an active state of the tongue (Gerard et al. 2005). A more recent publication showed that the stiffness of the tongue in rest might be four times less (Kappert et al. 2021). Moreover, hyperelastic material models used in most FE tongue models cannot cover all the complex properties of the tongue (Hermant et al. 2017). In this study, the FE model became unstable in extreme positions using lower stiffness values. Because the same mechanical and muscle properties are used for all personalized models, we assumed that the relative difference between models could still be used to analyze the effect of personalizing the muscle bundles of the FE model. However, mechanical properties were not the only limiting factor. Also, the specific muscle morphology obtained from CSD MRI contributed to the small magnitude of motion that is smaller than other non-personalized models in the literature that use the same mechanical properties (Buchaillard et al. 2009; Hermant et al. 2017; Kappert et al. 2019b). Manual editing of the muscle morphology might improve the magnitude of motion, but it was not considered as it would compromise the goal of this research, which was to automate FE modeling based on CSD data. Finally, the scaling of the predicted ROMs was, although very close, not the same in every movement direction. In part, this can also be contributed to the material properties which can have a different impact on the deformation in for example a down and a left movement. The difference between left and right, however, corresponded to an asymmetry in the measured ROM, specifically a deviation to the left. As explained previously (Kappert et al. 2019a), this may have been caused by the order of instructions given by the investigator.

In conclusion, we demonstrated that biomechanical models based on CSD MRI contribute significant to the personalization of biomechanical models. To our knowledge, we are the first to report this personalization step for improving the prediction of tongue mobility. Additional research is needed to improve the performance of biomechanical models to match the same magnitude of motion as a real tongue. In the future, personalization may improve other biomechanical models such as those of speech and swallowing, potentially leading to better simulations of actual tongue functionality. In rehabilitation after tongue cancer surgery, models can potentially be used to simulate the tongue function that could be regained by practice. In the preoperative setting, we would expect an even larger potential for the prediction of tongue function, as alterations in tongue shape and musculature due to tumor growth would also be accounted for. Therefore, these results harbor a promising perspective for the development of biomechanical models that would better predict function loss of oral cancer patients and thus improve the choice of treatment in these patients.

## Data Availability

Upon request.

## Appendix I

To avoid manual manipulation of the tracts in the atlas model, four filtering steps were used. A filter was only applied if it contributed to removal of faulty tracks. The first filter checked for deviations in the tracts from a global angle. This only worked well for muscle bundles with one global direction. The next filter calculated the angle within a single track. This did not work well with curved muscles like the superior longitudinal muscle. The third filter checked for the number of neighbors and at a certain distance, thus removing tracks that are too far from the rest. The alpha shape controlled the curvature of the convex hull that enclosed a muscle bundle and is explained in the MATLAB documentation (https://nl.mathworks.com/help/matlab/ref/alphashape.html).

**Table:**

	The angle of the vector cannot deviate more than …° from the total mean vector direction of all tracks	The angle of the vector cannot deviate more than …° from the total mean vector direction of one muscle track	Tracks with less than … neighbors at a distance of … mm will be removed	Alpha shape
Vertical	45 (*z*-axis)	45	3/0.03	0.011
Transverse			5/0.20	0.03
Superior longitudinal			6/0.20	0.014
Mylohyoid			5/0.05	0.015
Inferior longitudinal	45 (*y*-axis)	45	5/0.04	0.011
hyoglossus		45	3/0.04	0.011
Geniohyoid			3/0.02	
Genioglossus		45	10/0.10	0.011
Digastricus		45	3/0.03	0.011

## Code availability

Upon request.
